# Aspiration-sclerotherapy versus laparoscopic de-roofing in the treatment of renal cysts: which is better?

**DOI:** 10.1186/s12882-020-01832-7

**Published:** 2020-05-24

**Authors:** Xueling Zhang, Dehong Cao, Peizhen Han, Zhengju Ren, Jia Wang, Qiang Wei

**Affiliations:** 1 Department of Urology, West China Hospital, West China Hospital, Sichuan University, No. 37, Guoxue Alley, Chengdu, Sichuan People’s Republic of China; 2 State Key Laboratory of Biotherapy and Cancer Center, West China Hospital, Sichuan University, Chengdu, 61000 People’s Republic of China

**Keywords:** Aspiration-sclerotherapy, Laparoscopic de-roofing, Renal cysts, Meta-analysis

## Abstract

**Background:**

To compare the clinical efficiency between aspiration-sclerotherapy (AS) and laparoscopic de-roofing (LD) in the management of renal cysts through meta-analysis of comparative studies.

**Method:**

A comprehensive literature search was performed by PubMed, MEDLINE, Ovid and Web of Science for relevant studies published up to January 2020. The statistical analyses were conducted with Review Manager 5.3.0 and Stata 15.1. The sensitivity analysis was also carried out to confirm the reliability of this Meta-analysis.

**Results:**

Our searches of literature generated 6 studies (1547 patients incorporated) comparing AS with LD in the impacts of renal cyst therapy. Of these, 6 studies contained 1106 and 441 patients who were treated with AS and LD, respectively. The outcome of this meta-analysis indicated that LD group was superior in symptomatic successful rate [Odds Ratio (OR): 0.28; 95%Confidence Interval (CI): 0.09 to 0.86; *P* = 0.03), radiological successful rate (OR: 0.06; 95%CI: 0.02 to 0.15; *P* < 0.01) and recurrence rate (OR: 6.08; 95%CI: 2.81 to 13.15; *p* < 0.01). Nevertheless, AS group had shorter treatment time [Mean Difference (MD):-51.10; 95% CI:-73.01 to − 29.20; *p* < 0.01]. No statistically significant difference was showed in the rate of complications (OR: 3.19; 95% CI: 0.39 to 25.88; *P* = 0.28).

**Conclusions:**

In our meta-analysis, LD had higher symptomatic successful rate, radiological successful rate as well as lower recurrence rate than AS, while the treatment time was longer.

## Background

The renal cyst is one of the most common diseases among kidney illnesses and age, hypertension, proteinuria as well as microscopic hematuria are regarded as risk factors of that [1]. In an adult health-screening cohort, Ozveren et al. [2] found that the prevalence of renal cysts was 7.7% under ultrasonography,besides, greater numbers of renal cysts were found in males. Moreover, Kong et al. [3] managed a large sample cross-sectional study about renal cysts, whose results showed the prevalence of it among Chinese adults was 10.5% and renal cysts were significantly correlated with renal damage: the estimated glomerular filtration rate (eGFR) of 5.7% patients who with more than one renal cyst was decreased under 60 ml/min/1.73m^2^. Nowadays, according to the morphological description of renal cysts, cysts of Bosniak III, IV are symptomatic and even malignant to some extent [4]. Referring to the guidelines of the European Association of Urology (EAU), renal cysts (Bosniak III, IV) are recommended to be treated through surgery [5]. Therefore, we should pay attention to the treatments of renal cysts in particular.

Aspiration-sclerotherapy (AS) and laparoscopic de-roofing (LD) are main therapies for renal cysts [6]. LD is usually applied to large compressive cysts or some cysts keeping increasing in size rapidly, while AS takes an advantage in time-consuming and cost-saving but its reliability has not yet been guaranteed [7]. Based on the current clinical literature, we first conducted this meta-analysis to compare AS with LD in the management of renal cysts.

## Methods

### Search strategy

A systematic and comprehensive search was performed in PubMed, Ovid, EMBASE and Web of Science by two reviewers independently. “sclerotherapy”, “aspiration”, “laparoscopy”, “laparoscopic”, “de-roof”, “decortication”, “decompression”, “ablation”, “kidney cysts”, “renal cysts” are used as search terms to seek for relevant studies published up to January 2020. We followed the guideline of Preferred Reporting Items for Meta-Analyses [8].

### Inclusion criteria

- Clinical experimental articles (both prospective and retrospective experiments)
- Patients with renal cysts (Bosniak III, IV)
- Studies comparing AS and LD in the treatment of renal cysts only.
- Articles with full-text.

### Exclusion criteria

- Studies addressing AS only or LD only.
- Studies comparing AS and LD among patients with other kidney diseases but not renal cysts.
- Systematic review articles.
- Academic conference abstracts without full-text.

### Date extraction

Data extraction and quality assessment of included studies were performed by two independent reviewers. The following information was selected from each included study: first author’s name, publication year, country, type of treatments, sample number, age, gender, diameter of cyst and length of follow-up. We also extracted some particular outcomes from each treatment type such as clinical presentation, rate of symptomatic success, rate of radiological success, time of treatment, rate of complications and rate of recurrence. In the end, all the statistics were divided into two groups according to the way of therapy for renal cysts and compared accurately.

### Quality assessment

Criteria provided by the Oxford Centre for Evidence-Based Medicine was used to assess the level of evidence for each study. The Newcastle–Ottawa Scale was used for the estimation of these studies` methodological quality [9]. Two reviewers accomplished the appraisal respectively. Disagreements were solved after discussions. If disagreement persisted, a third reviewer would participate in the discussion until an agreement was reached.

### Statistical analysis

This meta-analysis compared symptomatic successful rate, radiological successful rate, treatment time, rate of complications and recurrence rate between AS and LD during the treatment of renal cysts. Review manager software version 5.3.0 and Stata 15.1 were used to evaluate the outcomes of each study for comparison. Odds ratio (OR) and mean difference (MD) were used to estimate binary variables and continuous variables separately. If outcomes from these 6 studies were shown with 95% confidence interval (CI) only, the standard deviation (SD) was calculated by the statistical calculation declaimed by Hozo and colleagues [10]. A random-effect model was used in this Meta-analysis. Heterogeneity of these researches was evaluated using the chi-square test and I^2^ > 50% was regard as high heterogeneity. A *p*-value < 0.05 was considered as statistically significant. Sensitivity analysis was also performed to evaluate the reliability of the data. The pooled ORs were assessed by removing 1 study each time to estimate the effects of an individual study.

## Results

### Study characteristics

**Fig. 1:**
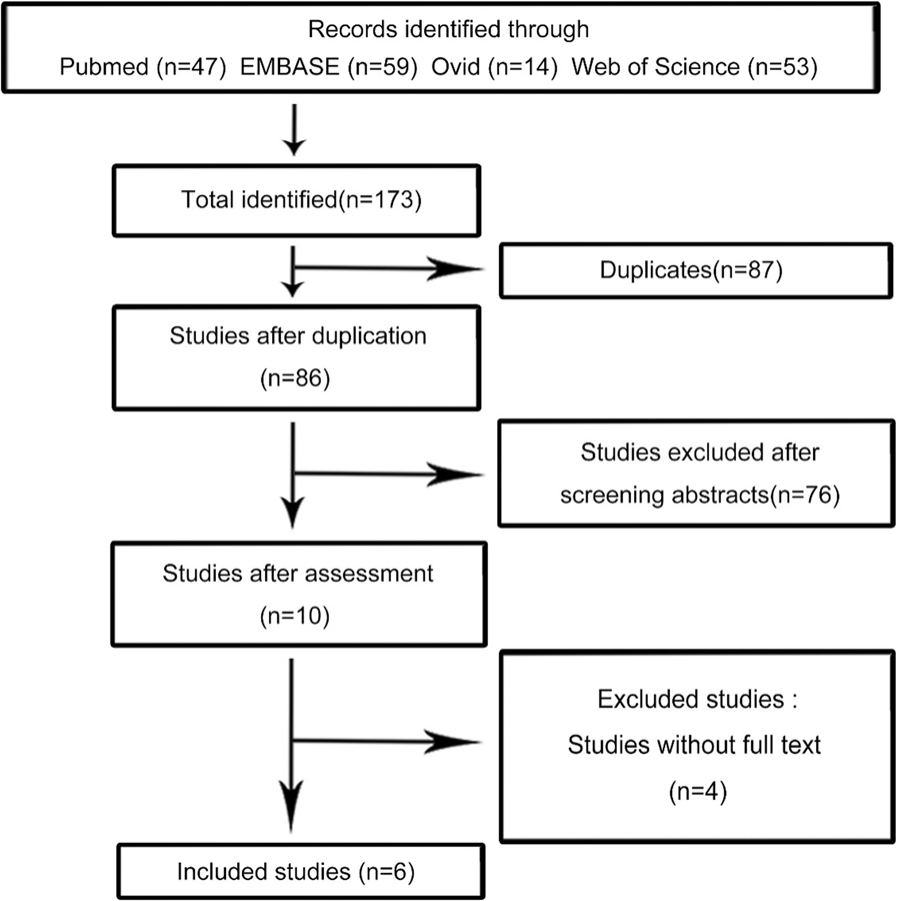
Flow chart of the strategy of search and selection

**Fig. 2:**
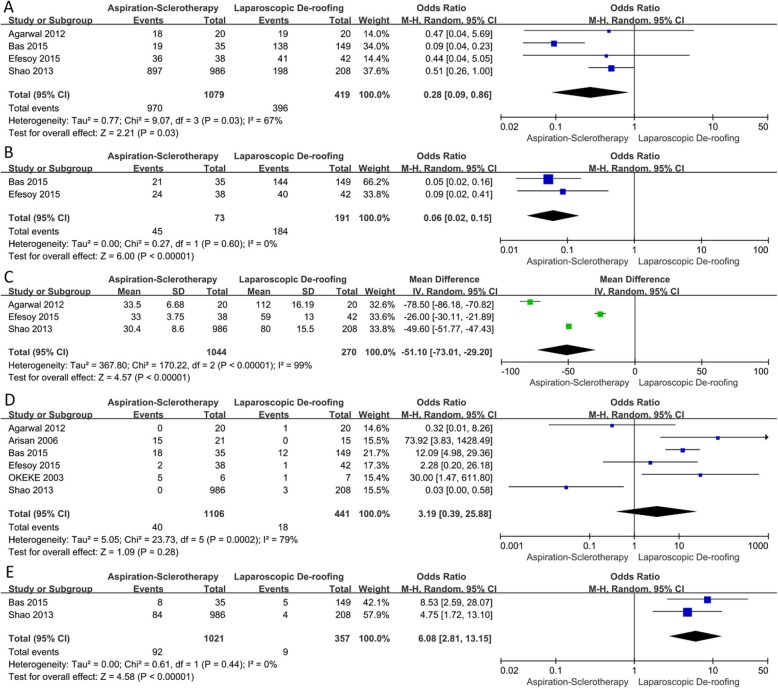
Forest plot [**a**. symptomatic successful rate; **b**. radiological successful rate; **c**. treatment time (minutes); **d**. complications; **e**. recurrence]

Our researches of literature generated 6 studies [11–16] comparing AS with LD in the impacts of renal cysts treatment. These studies contained 1106 and 441 patients who were treated with AS and LD respectively. Characteristics (including first author’s name, publication year, country, type of treatments, sample number, age, ratio of male, diameter of cyst and length of follow-up) and quality assessment of all studies were summarized in Table 1. Meta-analysis results of these 6 studies are presented in Fig. 2. Flow chart diagram demonstrating the strategy of search and selection was shown in Fig. 1.

**Table 1:** The characteristics of patients included

**Autho**r	**Country**	**Treatment**	**Sample (n)**	**age (y/o)**	**Male ratio (%)**	**Mean diameter o cyst (cm)**	**Clinical presentation (n)**				**Follow up (m)**	**NOS**
							**pain**	**lump**	**Hypertension**	**Hematuria**		
**Efesoy** [11] **2015**	**Turkey**	**AS**	**38**	**53.2 ± 13.7**	**57.9**	**9.5 ± 2.9**	**36**				**6**	**7**
		**LD**	**42**	**51.1 ± 11.6**	**54.8**	**8.5 ± 2.7**	**38**					
**Bas** [12] **2015**	**Turkey**	**AS**	**35**	**59**	**62.8**	**7.2**	**21**	**7**	**3**	**4**	**35**	**7**
		**LD**	**149**	**57.7**	**56.8**	**7.9**	**119**	**16**	**5**	**9**		
**Shao** [13] **2013**	**China**	**AS**	**986**	**55.3 ± 14.7**	**53.2**	**6.3 ± 1.4**	**494**		**41**	**61**	**12**	**7**
		**LD**	**208**	**46.8 ± 11.9**	**41.8**	**7.5 ± 2.3**	**101**		**19**	**16**		
**Agarwal** [14] **2012**	**India**	**AS**	**20**	**46.4 ± 9.48**	**60**	**6.58 ± 0.85**	**10**	**4**		**2**	**3**	**8**
		**LD**	**20**	**44.8 ± 14.28**	**50**	**7.28 ± 0.94**	**9**	**3**		**1**		
**Arisan** [15] **2006**	**Turkey**	**AS**	**21**	**60**	**52.4**	**6.0**	**21**		**4**		**12**	**8**
		**LD**	**15**	**57**	**40**	**9.0**	**15**					
**OKEK E** [16] **2003**	**UK**	**AS**	**6**	**50.8**	**33.3**	**6.0**	**5**	**1**			**17**	**7**
		**LD**	**7**	**50.9**	**28.6**	**8.0**	**7**					

*AS* aspiration-sclerotherapy

*LD* laparoscopic de-roofing

*NOS* Newcastle–Ottawa Scale

### Quality assessment

The evidence levels of all the studies were rated Level 2 according to the Oxford level of evidence criteria. The studies written by Agarwal et al. [14] and Arisan et al. [15] were scored eight stars based on the Newcastle–Ottawa Scale. The rest of studies were scored seven stars each.

### Meta-analysis and sensitivity analysis

#### Symptomatic successful rate

**Fig. 3:**
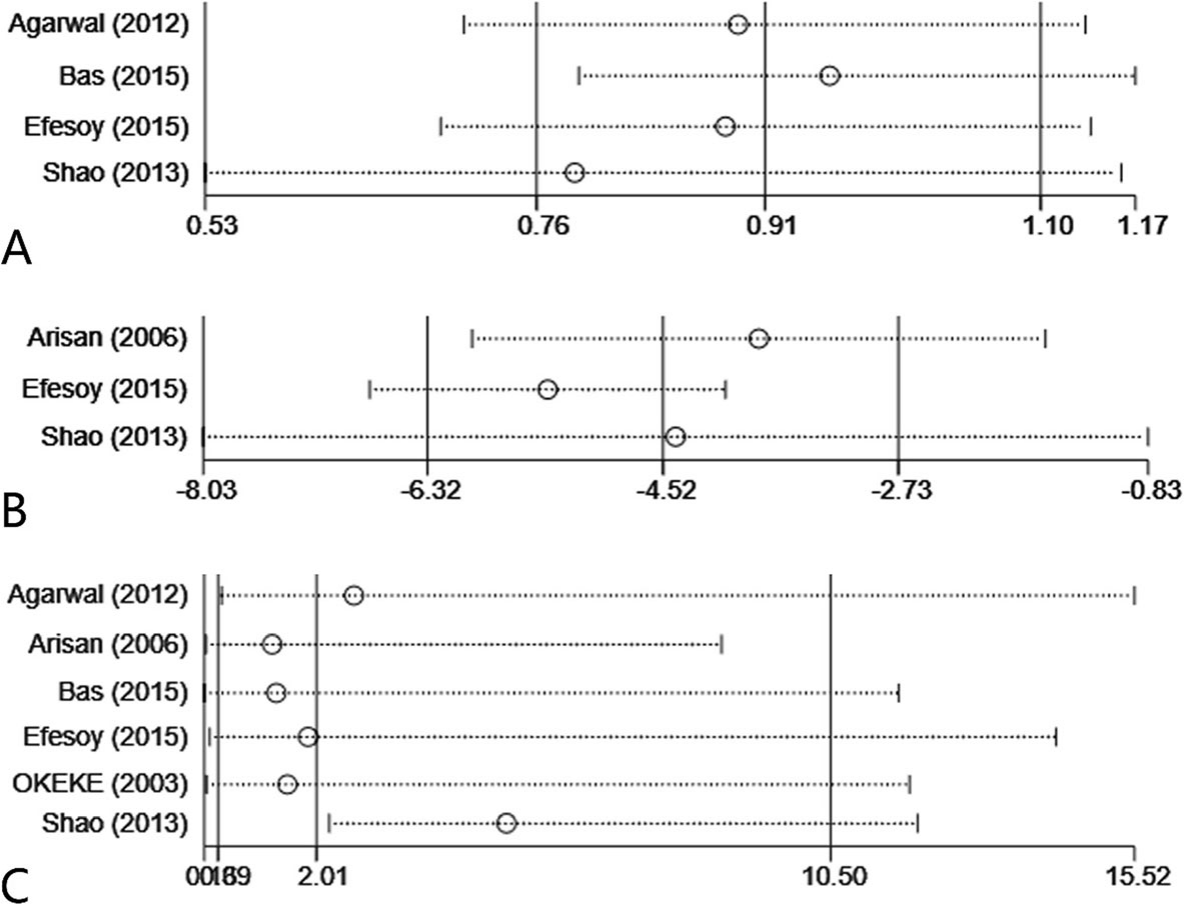
Sensitivity analysis (**a**. symptomatic successful rate; **b**. treatment time; **c**. complications)

Four studies [11–14] involving 1498 patients compared the cure rate of symptom between AS group and LD group. 396 patients (419 patients in total) got symptomatic cure through LD therapy, while 970 patients (1079 patients totally) recovered after treated by AS. The result of this meta-analysis showed that LD group had higher rate of symptomatic success (random effect model; OR: 0.28; 95% CI: 0.09 to 0.86; *P* = 0.03) with a slightly high heterogeneity (*P* = 0.03; I^2^ = 67%; Fig. 2a). The sensitivity analysis of this outcome was depicted in Fig. 3a, which demonstrated that the pooled OR was not influenced by any study from these 4 researches.

#### Radiological successful rate

Two studies [11, 12] including 264 patients compared the successful rate of radiology between AS group and LD group. 184 patients (among 191 patients) had radiological healing by LD, and 45 patients (73 patients in total) got cured by AS. A meta-analysis of these studies stated that LD group is significantly higher in the matter of radiological cure rate compared with AS (random effect model; OR: 0.06; 95%CI: 0.02 to 0.15; *P* < 0.01). Statistical heterogeneity was not illustrated in the pooled analysis (*P* = 0.60; I^2^ = 0%; Fig. 2b).

#### Treatment time

Three studies [11, 13, 14] involving 1314 patients compared the treatment time between AS group and LD group. The time of AS therapy was 30.55 min on average, comparing to that of LD was 79.10 min. The result of meta-analysis showed that AS was associated with a significantly lower time-consuming in the therapy procedure (random effect model; MD:-51.10; 95% CI:-73.01 to − 29.20; *p* < 0.01), but the heterogeneity of it was high (*P* < 0.01; I^2^ = 99%; Fig. 2c). The sensitivity analysis about this result was shown in Fig. 3b, which declared the pooled OR was not affected by any study from these 3 articles.

#### Complications

Six studies [11–16] containing 1547 patients compared the number of complication events between AS group and LD group. Details of complications were delineated in Table 2. The outcome of our meta-analysis illustrated that no statistically significant difference was found between these two groups (random effect model; OR: 3.19; 95%CI: 0.39 to 25.88; *P* < 0.01) with a moderately high heterogeneity (P < 0.01; I^2^ = 79%; Fig. 2d). The sensitivity analysis on complications was also performed and presented in Fig. 3c, proving the pooled OR was not influenced by any study from all the 6 articles.

**Table 2:** Complications

**Author**	**Complications**	**Pain**	**Hypertension**	**Hemorrhage**	**Fever**	**Port Infection**	**Total**
**Efesoy** [11] **2015**	**AS**				**2**		**2**
	**LD**			**1**			**1**
**Bas** [12] **2015**	**AS**	**15**	**3**				**18**
	**LD**	**7**	**4**	**1**			**12**
**Shao** [13] **2013**	**AS**						**0**
	**LD**					**3**	**3**
**Agarwal** [14] **2012**	**AS**						**0**
	**LD**					**1**	**1**
**Arisan** [15] **2006**	**AS**	**15**					**15**
	**LD**						**0**
**OKEKE** [16] **2003**	**AS**	**5**					**5**
	**LD**			**1**			**1**
**Total**		**42**	**7**	**3**	**2**	**4**	**58**

*AS* aspiration-sclerotherapy

*LD* laparoscopic de-roofing

#### Recurrence

Two studies [12, 13] including 1378 patients compared the number of recurrence events of AS group with that of LD group. Our meta-analysis of these studies illustrated that LD was significantly associated with lower rate of recurrence compared with AS (random effect model; OR: 6.08; 95%CI: 2.81 to 13.15; *p* < 0.01). Statistical heterogeneity was not stated in this meta-analysis (*P* = 0.61; I^2^ = 0%; Fig. 2e).

## Discussion

Currently, comparisons of the effects between AS and LD in the treatment of renal cysts were lacking, therefore, we first conducted a meta-analysis on this subject. Statistics in Table 1 show that renal pain would be the primary presenting symptom among patients with renal cysts. In addition to this, renal lump, hypertension and hematuria frequently happened to renal cyst patients as well according to Table 1. Besides, Kim et al. [17] investigated 3249 patients with renal cysts and they also proved that the occurrence of renal cysts was positively related to hypertension, which has the following characteristics: bilateral distribution, number of cysts no less than two and diameter of cysts larger than 1 cm. Although renal cyst is a benign disease in most cases, complicated variations of that still has the connection of renal cell carcinoma, which may urge patients with renal cysts to be more cautious about the importance of regular follow-up [18].

At present, treatments such as AS and LD are available to renal cyst patients. Relating to AS, it is time-saving, economical, well tolerated and technique-simplifying so that can be performed under local anesthesia and even in the outpatient rooms in most cases [19]. Our meta-analysis also illustrated that treatment time of AS is significantly shorter than LD. As we all know, AS can be conducted under local anesthesia while LD should be operated with general anesthesia. Thus, LD needs more time for therapy. The high heterogeneity of treatment time may be caused by different ways of records about these data. Some of included studies collected the entire time of hospital stay, nevertheless, the rest of studies only recorded the operation time, which also affected the heterogeneity.

Studies reported by Ali et al. [20] and Monville et al. [19] evaluated the efficiency of ultrasound-guided AS treatment among patients with renal cysts, their results showed that the radiological successful rate were up to 98 and 91.6% respectively meanwhile the complication and recurrence leveled off to zero. However, the rates of radiological success reported by Efesoy et al. [11] and Bas et al. [12] were 63.2 and 60% separately, which were also presented in Table 2. Ethanol contacting the cyst wall causes protein degeneration, cell death as well as inflammatory fibrosis so that patients should keep 5 to 10 min at least in each position according to the diverse cyst size and volume [21]. Therefore, the difference among the radiological cure rates might be ascribable to the different treatment procedure, which also would be hard to make a standard therapy. Referring to the research of Dell’Atti at al [22]., polidocanol, whose successful outcome ratio was significantly higher (90% vs. 61%) while failure ratio was significantly lower (3% vs. 33%) than traditional sclerosing agent (ethanol), could be a better choice of AS therapy.

In addition to this, although study reported by Zhong et al. [23] shows no recurrence was observed after AS treatment, in the outcome of our meta-analysis, it was remarkably higher in AS group than LD group in the matter of recurrence. The reason why simple fluid aspiration was ineffective and even promoted the recurrence of cyst could be that the renal cyst epithelium was not destroyed by sclerosing agents completely and adhered to each other, thus the remained cyst wall can still secrete fluid [24].

In terms of LD therapy, it has the advantage in high rate of cure and low rate of recurrence, and it can be thought as a complete treatment of renal cysts [25]. Nasseh et al. [26] collected the data of renal cyst patients treated with LD in their center and 91.3% patients reached symptomatic and radiological success while only one patient got recurrence, which was consistent with our meta-analysis results. As LD operation preferred to excising the complete cyst including all cyst walls, therefore, the left tissues were out of secreting function, which led to high cure rate as well as low recurrence rate [27]. Hence, LD could be the standard in the management of renal cysts, especially suited for patients failed after AS therapy [28].

To enhance the efficiency of LD treatment, Lai and colleagues [29] studied the impacts of perirenal pedicled fat tissue wadding technique (PPFTWT) on the recurrence rate during this surgery operation, they found that LD using PPFTWT can decrease the rate of cyst recurrence evidently. Inserting fat tissue into the cavity of the cyst and fixing it prevented the cyst wall from adhering to the residuary cyst wall or surrounding tissue, and contributed to the secretion drainage and absorption of the remaining cyst wall, thus declined the risk of cyst recurrence [30]. Therefore, LD with PPFTWT may be a fitful way in the treatment of renal cysts.

When it comes to complications, our statistics showed there was no significant difference between AS group and LD group, both of which can cause post-treated complications such as fever, infectious, pain, hemorrhage. On account of ethanol as the common sclerosing agents in AS treatment, patients might get alcohol intoxication and lose consciousness even injury femoral nerve due to the rupture of cysts treated by AS [31]. As for LD therapy, vessels damage and subcutaneous emphysema might happen to patients during the process of cyst ablation and establishing pneumoperitoneum [32].

The limited included studies in our meta-analysis and the heterogeneity of some date were two main limitations of this study. Due to the lack of researches on the comparations between AS and LD, we included six articles merely and the patient selection bias, heterogeneity of cyst diameter and follow up time or difference of operator training/experience could also be limitations. Therefore, further studies are expected to confirm our outcomes. As for the heterogeneity, sensitivity analysis was conducted and delineated in Fig. 3. In the study of Bas et al. [12], the difference of treatment techniques may contribute to the heterogeneity in symptomatic successful rate. Besides, in terms of complication rate, the researches of Agarwal et al. [14] and Shao et al. [13] only recorded some severe complications such as port infections so that they might omit some information about complications after aspiration treatment, which also brought about the heterogeneity.

## Conclusions

Summarily, it is the first meta-analysis that compared the effects between AS and LD in renal cyst treatment. AS took the advantage of less time-consuming, few potential injuries caused by surgery and the demand of local anesthesia only, which was more suitable for elder people with renal cysts. Nevertheless, higher symptomatic cure rate, radiological cure rate and lower rate of recurrence are the characteristics of LD. Consequently, patients younger or seeking for complete regression were recommended to give priority to LD therapy. Due to the limited comparative studies and heterogeneity of some statistics, more researches are needed to validate our conclusion.

## Data Availability

The datasets used and/or analysed during the current study are available from the corresponding author on reasonable request.

## Abbreviations

eGFR: The estimated glomerular filtration rate
EAU: European Association of Urology
AS: Aspiration-sclerotherapy
LD: Laparoscopic de-roofing
OR: Odds ratio
MD: Mean difference
CI: Confidence interval
SD: Standard deviation
